# Multiplexed Immunohistochemistry for Molecular and Immune Profiling in Lung Cancer—Just About Ready for Prime-Time?

**DOI:** 10.3390/cancers11030283

**Published:** 2019-02-27

**Authors:** Paul Hofman, Cécile Badoual, Fiona Henderson, Léa Berland, Marame Hamila, Elodie Long-Mira, Sandra Lassalle, Hélène Roussel, Véronique Hofman, Eric Tartour, Marius Ilié

**Affiliations:** 1 Laboratory of Clinical and Experimental Pathology, Hospital-Integrated Biobank (BB-0033-00025), Nice Hospital University, FHU OncoAge, Université Côte d’Azur, Nice 06000, France; hofman.p@chu-nice.fr (P.H.); leaberland370@gmail.com (L.B.); hamila.m@chu-nice.fr (M.H.); long-mira.e@chu-nice.fr (E.L.-M.); lassalle.s@chu-nice.fr (S.L.); hofman.v@chu-nice.fr; ilie.m@chu-nice.fr (V.H.); 2 Team 4, Institute for Research on Cancer and Aging, Nice (IRCAN), INSERM U1081/UMR CNRS 7284, FHU OncoAge, Université Côte d’Azur, Nice 06107, France; 3 Department of Pathology, Hôpital Européen Georges Pompidou, APHP, Paris 75015, France; cecile.badoual@aphp.fr (C.B.); helene.roussel@aphp.fr (H.R.); 4 INSERM U970, Université Paris Descartes Sorbonne Paris-Cité, Paris 75015, France; eric.tartour@aphp.fr; 5 Department EMEA, Indica Labs, 2469 Corrales Rd Bldg. A-3 Corrales, NM 87048, USA; fhenderson@indicalab.com; 6 Department of Immunology, Hôpital Européen Georges Pompidou, Paris 75015, France

**Keywords:** multiplexed, brightfield, chromogenic, fluorescence, molecular, immune profiling, immune-oncology, digital, lung cancer

## Abstract

As targeted molecular therapies and immuno-oncology have become pivotal in the management of patients with lung cancer, the essential requirement for high throughput analyses and clinical validation of biomarkers has become even more intense, with response rates maintained in the 20%–30% range. Moreover, the list of treatment alternatives, including combination therapies, is rapidly evolving. The molecular profiling and specific tumor-associated immune contexture may be predictive of response or resistance to these therapeutic strategies. Multiplexed immunohistochemistry is an effective and proficient approach to simultaneously identify specific proteins or molecular abnormalities, to determine the spatial distribution and activation state of immune cells, as well as the presence of immunoactive molecular expression. This method is highly advantageous for investigating immune evasion mechanisms and discovering potential biomarkers to assess mechanisms of action and to predict response to a given treatment. This review provides views on the current technological status and evidence for clinical applications of multiplexing and how it could be applied to optimize clinical management of patients with lung cancer.

## 1. Introduction

Lung cancer is the leading cause of cancer death among males, and the second most common among females worldwide [1]. Approximately 80% of newly diagnosed patients with non-small cell lung cancer (NSCLC) have unresectable locally advanced or metastatic disease [2]. In these patients, current treatment strategies, across all lines of therapy, include chemotherapy regimens based on histology, targeted drugs for patients carrying specific genomic alterations and immunotherapy using immune checkpoint inhibitors (ICIs), in particular monoclonal antibodies targeting programmed cell death-1 (PD-1) and programmed cell death ligand-1 (PD-L1) [3,4,5,6,7]. The development of molecularly targeted therapies, as well as ICIs, has improved outcomes in the metastatic setting for NSCLC patients who harbor somatically activated oncogenes such as *EGFR* and *BRAFV600,* rearranged *ALK* or *ROS1,* or PD-L1 expression ≥50% of tumor cells [3,4,5]. However, even with these molecular strategies, a large proportion of patients do not attain prolonged disease control, and the 5-year survival rate does not exceed 5% [8,9,10].

Patients with suspected stage IIIB/IV NSCLC require tissue or cytology sampling to confirm the diagnosis (e.g., adenocarcinoma vs. squamous cell carcinoma vs. other lung histological subtypes), as this determines eligibility for biomarker testing and further therapeutic strategies [11]. Several immunohistochemical (IHC) markers (e.g., TTF1, p40, INSM1) may be needed to confirm and subtype lung carcinoma [12,13]. Additional tumor material is required for interrogating predictive biomarkers, using IHC (e.g., ALK, ROS1, PD-L1), in situ hybridization (ISH; e.g., ALK, ROS1) or sequencing techniques (e.g., *EGFR*, *BRAF* V600E, etc.). Moreover, in the context of precision oncology, lung cancer patients may be enrolled in ongoing clinical trials (https://clinicaltrials.gov/) and tumor samples may be used for basic and clinical research studies [14]. 

For these procedures, sufficient material of high quality is mandatory. In a large number of cases, the tumor material on which all diagnostic and predictive test must be theoretically be performed might be sparse, containing only a small number of tumor cells [15]. Small biopsy samples with few tumor cells might often only allow diagnosis and classification of tumor subtype, and additional tests may be compromised [11,15].

In the current boost to improve the tailored approach to the clinical management of patients with NSCLC, pathologists and researchers deal continuously with an unresolved dilemma for exploring a growing number of protein biomarkers on small-sized tumor samples. In this context, multiplexed immunohistochemistry (mIHC) has recently emerged as a potent tool for the simultaneous detection of multiple protein biomarkers on the same tissue section to expand the molecular and immune profiling of NSCLC, while preserving tumor material. Over the last years, the role of IHC has been constantly extended to improve diagnosis, and to guide prognosis and treatment of NSCLC patients, while requiring assessment of an increasing number of protein targets. In addition, multiplying serial tissue sections to stain for a single marker per slide, can waste small biopsy specimens, entangle the correlation of section-to-section protein expression, and leave insufficient tumor material for additional analyses [16]. Multiplexing can be carried out using chromogenic or fluorescent staining methods. Complex fluorescent multiplexing systems are currently being developed (reviewed in this Special Focus by Parra et al.) [17]. New approaches compatible with high levels of target multiplexing and suitable for use on formalin-fixed paraffin-embedded (FFPE) samples have recently demonstrated the potential to be transferred to the clinical setting [18,19,20,21,22]. For instance, direct simultaneous assessment by mIHC of both immune and tumor-related pathways and their spatial relationships, in a single tissue sample, may empower more accurate patient stratification for immunotherapy [23]. 

Finally, in recent years, mIHC technology has seen rapid advancements in image acquisition throughput, image resolution and data accuracy, allowing improvements in pathologist performance by automatically measuring parameters that are hard to achieve reliably by microscope, to extract comprehensive information on biomarker expression levels, co-localization, and compartmentalization. The present manuscript reports on mIHC approaches for molecular and immune profiling in lung cancer. 

## 2. Principles of Multiplexing Staining Methods

### 2.1. Chromogenic Multiplexed IHC 

Technical approaches of brightfield chromogenic mIHC include direct detection of antigens by primary antibodies from the same or different species that are directly labeled with different chromogens. Alternatively, an indirect mIHC detection method can be used with two or more layers of antibodies, allowing for increased amplification of signal [24]. The direct detection approach has several disadvantages, such as lower sensitivity for low abundance targets, the need for sizeable quantities of conjugated antibodies, which are usually more expensive, and the risk that antibody activity could be adversely affected by direct labeling [24]. The indirect approach can be limited by the number of available host species and the use of same species antibodies, which would thus require inactivation between successive cycles of immunolabeling [24]. 

The unwanted cross-reactivity between primary antibodies from different staining cycles is regarded as the main technical challenge in mIHC. The most frequent solution used to avoid such reactions is manual microwaving or heating of tissue slides to deactivate the preceding antibody [25,26]. Whereas microwaving is often used in research facilities when dealing with antibodies from the same host-species, it may not be an optimal method to be adopted in a routine clinical setting. Variable and heterogeneous results could be obtained by manual processing. Furthermore, microwaving can increase the damage of the tumor tissue and may remove small biopsies from the slides, especially if they have already been antigen retrieved by a previous heat-mediated procedure [27]. 

Another strategy for preventing cross-reactivity is the use of stripping buffers to elute the primary/secondary antibody complex [27,28]. A number of buffers with different pH, osmolality, detergent content and denaturing features were evaluated to strip the bound antibody complex from previous IHC staining cycles, however this produced variable results across studies. Certain buffers were found to be hazardous, to decolorize H&E stain and/or to reduce nuclear protein staining [27,28].

An alternative, more recent approach named “multiplexed immunohistochemical consecutive staining on single slide” (MICSSS), was developed for use on FFPE samples by applying repetitive cycles of immunoperoxidase labeling, image scanning, then chemical stripping of the chromogenic substrate [20,21]. However, this process can result in a labor-intensive protocol and a prolonged turnaround time to yield results that are not suitable for a routine clinical setting. Moreover, multiplexing may be limited due to tissue degradation after successive serial mIHC cycles [24,29]. 

**Figure 1:**
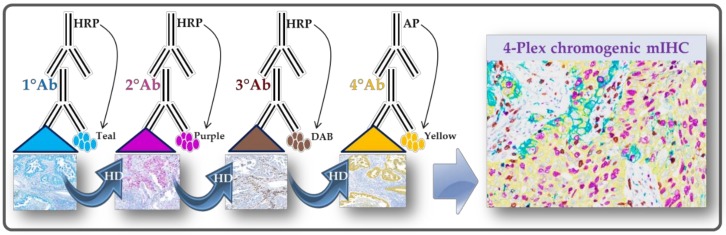
Chromogenic multiplexed immunohistochemistry assay scheme. The assay is using the sequential application of four unmodified primary antibodies with a specific heat deactivation (HD) step between staining cycles.

More recently, a fully automated mIHC technology using a thermochemical process (heat deactivation; HD) to deactivate an antibody complex between staining cycles on an automated slide stainer was first developed for fluorescent detection, and further applied to brightfield chromogenic detection (Figure 1) [30,31]. 

This technology allows the use of the first antibody from the same host species, detected by the anti-species secondary antibody conjugated to horseradish peroxidase (HRP). In the presence of its substrate, the active HRP, generates in-situ deposition of tyramide within the medium containing the chromogens. The bound primary antibody/secondary antibody complex is then eluted with a citrate/acetate buffer. Thus, the deposited chromogen-conjugated tyramide bounds covalently to the tissue near the first detected antigen. The same procedure is repeated to detect the following antigens [30,31]. Indeed, the sequential stripping may lead to wastage of the conjugated secondary antibodies, whereas the chromogen-conjugated tyramine remains stable by binding covalently to the electron-rich amino acids of detected proteins and by resisting to the elution with the stripping buffer [30,31]. Importantly, the automation allows for standardization of all critical mIHC steps, such as HD, reagent application, washing steps, control of temperatures, evaporation and humidity, while maintaining the integrity of the tissue architecture and the subsequent epitopes [32,33]. 

To setup a brightfield mIHC assay using sequential detection with unmodified primary antibodies and chromogenic detection, it is essential to optimize assay conditions on the tissue types of interest before testing clinical samples [34]. Thus, an optimal mIHC assay needs to assure several staining performances: (i) equivalent positive/negative signal to single “gold standard” IHC staining, (ii) robust dynamic proportion of low and high protein quantity, (iii) expected cellular staining topology (e.g., whole membrane, cytoplasmic, nuclear localization), and (iv) minimal overlap of chromogenic spectra for co-localized targets [34]. Recent developments have enabled optimal configurations suitable for testing on clinical samples. For instance, the order of chromogen deposition is determined by the effect of HD on each epitope, that is, the most HD-affected epitope is incubated first, with the least affected epitope incubated last. 

To offer the best detection sensitivity, other assay parameters must be taken into account such as the optimal epitope retrieval time to balance the signal/background ratio, and to protect the tissue architecture by optimizing the incubation time for each primary antibody [30]. Moreover, the number of antibodies for simultaneous immunolabeling on the same tissue slide has been extended up to six with the availability of additional chromogens [24,33]. In addition, a major technical challenge is the risk of insufficient deactivation of the primary antibody complexes, which could determine cross-reactions and may give false-positive signals. Besides efforts to optimize HD steps during assay validation, the imaging tools can help to anticipate or to detect potential cross-reactions [35].

### 2.2. Immunofluorescent Multiplexing

Many newly identified or discovered biomarkers, especially for cancer immunotherapy, are linked to the tumor microenvironment and need to be analyzed with new methodological tools. For years, it has become increasingly essential to develop staining and interpretation techniques for the different cell populations infiltrating or composing a tissue. This is particularly true in oncology. To date, as previously described, the use of immunohistochemistry can help the visualization of an antibody-antigen conjugation. It has been showed in the last subsection, that an antibody is conjugated to an enzyme, like a peroxidase, can catalyze a color-producing reaction. Alternatively, the antibody can also be tagged with a fluorophore. Nowadays the use of immunofluorescence is far easier due to technical improvement, like the use of stable fluorophores or the possibility to perform staining in paraffin embedded slides. Since years, research teams proposed immunoscoring, using single staining per slide, to identify prognostic factors [36]. However, the tumor microenvironment is too complex to be summarized by the exploration of a single marker. Chromogenic mIHC is one of the alternatives, and even if this technique is much easier to be used routinely, it is limited by the use of 4 antibodies on the same slide. In addition, fluorescence reveals membrane co-localizations (in the membrane or the nucleus), which is more difficult to obtain with the latter technique. Nevertheless, the use of multiple antibodies (mixed or used step by step) was restricted to the specificity of the primary and the risk of false positivity due to cross reactivity between them. Until recently, the single-parametric or even multiparametric (double or triple) staining, revealed by chromomeric or fluorescent staining, were most often read and interpreted directly by the researchers [37], with a lot of technical constraints. 

The microenvironment can now be studied using the multiplex fluorescence technique based on tyramide coupled to a fluorophore (e.g., Opal^®^, PerkinElmer, Waltham, MA, USA). This allows the simultaneous detection of several markers of interest on FFPE tissues. The concept of the technique is very close the one described above, the chromogenic mIHC assay using sequential application of four unmodified primary antibodies with a specific HD step between staining cycles. The main advantage of this technique is the multiplicity of the staining. The technique is based on a conventional fixation on the epitope of interest. The secondary antibody then binds to the primary antibody followed by Opal^®^ HRP polymer and one of the Opal® fluorophore adjunction. After deposition of Opal^®^ reagents, antibodies are stripped after use of a specific microwave to allow subsequent staining of other antigens. These cycles can be repeated at least seven to nine times. This seven to nine color multiplex staining technique makes it possible to more precisely characterize different cells and their interactions with their environment, on the same paraffin slide [38,39]. However, the use of these new techniques requires the acquisition of specific expertise for in situ multiple staining. Automation of this different process is now efficient and several autostainers are able to execute most of the steps previously described.

**Figure 2:**
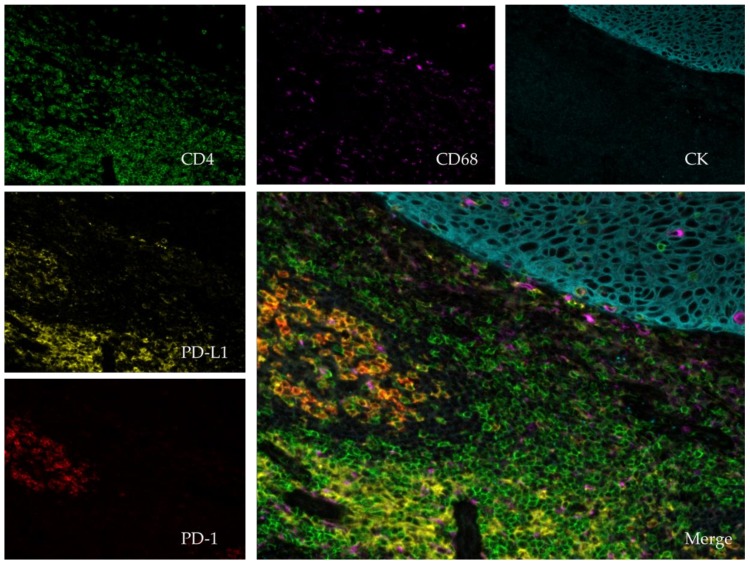
Immunofluorescent multiplexing, image scanned with a spectral scanner (Polaris^®^; PerkinElmer, Waltham, MA, USA) using 20× magnification. The tissue is a paraffin embedded tonsil. The stains are as follows: pan-Cytokeratin (CK, teal), CD4 (green), CD68 (purple), PD-1 (red), PD-L1 (yellow) and dapi (blue). The central picture compiles the entire staining (merge).

For the validation of the different panels of multiparametric IHC markers, in particular for the exploration of the immune system, staining can be performed on tonsil tissue sections as this contains lympho-epithelial structures (Figure 2). Before any application on a cohort, especially when it concerns lung sections, the validation of staining on pulmonary tissue sections as a positive control is highly recommended. In addition, the same positive tissue control could be run on the same slide tested with mIHC, such as is currently performed for clinical diagnostic IHC.

The principle of a multiplex analysis of the tumor microenvironment is the automatic acquisition of a large surface, or the entire slide, quickly and sustainably. Having a fast acquisition time (milliseconds for each illuminated spot) is fundamental for fluorescence techniques because it prevents the "bleaching" which is the progressive extinction of the fluorescent signal after excitation.

## 3. Clinical and Translational Research Applications: Brief Literature Review and Own Results 

Despite the impressive recent achievements in therapeutic strategies for NSCLC treatment, clinical responses have remained limited to subsets of patients, relapse has occurred in the vast majority of patients, and only few effective predictive biomarkers have been defined [40]. The development of more effective predictive biomarkers is needed to optimize patient benefits, minimize the risk of toxicities, and guide combinatorial approaches. In particular, the emerging picture in immune-oncology requires a comprehensive understanding of the tumor microenvironment that is the immune landscape of NSCLC, which results from a complex dynamic cross-talk between the tumor and the immune system [23,40]. Current efforts on novel biomarker candidates include research on identification and quantification of different immune cell subsets, their spatial localization and relationships within tumor areas, the expression of different immune checkpoint markers, tumor mutational burden, and immune gene signatures [23,40]. Thus, the complete picture will be generated by the integrative high-dimensional analysis of the tumor and immune profile based on multiple technological approaches, including mIHC [23]. 

### 3.1. Chromogenic Multiplexed Immunohistochemistry 

The MICSS technology has demonstrated that high-dimensional characterization of the immune contexture before and after treatment with ICIs correlates with response to treatment in cancer patients [20,21]. The immune contexture describes the density, localization, and organization of the immune cells within solid tumors [41]. By analyzing the composition of complex immune cell populations, the neutrophil/dendritic cell density score refined the prognostic value of tumors rich in T-cells and was an independent marker of outcome in NSCLC patients [21]. 

Another MICSS mIHC platform with computational image processing workflows, including image cytometry, enabled simultaneous evaluation of three 12-antibody biomarker panels in one FFPE tissue section, highlighting the impact of in situ monitoring of immune complexity for patient stratification to improve biomarker discovery and development [20]. The diverse immune complexity within lymphoid- or myeloid-inflamed tumors as detected by this platform, correlates with clinical outcomes and tumor sub-classification in head and neck squamous cell carcinoma. In addition, myeloid-inflamed and T cell exhaustion status correlated with shorter overall survival and the therapeutic response to vaccination therapy in patients with pancreatic ductal adenocarcinoma [20].

Recently, a chromogenic mIHC method revealed that a high density of tumor-associated neutrophils (TANs), but not stromal TANs, may have a divergent prognostic effect in NSCLC, negative in adenocarcinomas, while in squamous cell carcinoma it is a good prognostic factor [42]. Overall, the in situ high-dimensional assessment of immune cells reveals the potential of mIHC to expand the immunoscore in NSCLC patients in a clinically relevant manner [43,44,45]. 

Interestingly, a recent clinical trial has supported the role for neoadjuvant immunotherapy in surgically resectable NSCLC, suggesting that the neoadjuvant regimen may lead to early induction of an adaptive anti-tumor immunity, which could be responsible for preventing distant metastases [6]. While this treatment strategy is still in an early stage of clinical development, there are several pending questions that are yet to be answered, including whether the major pathologic response could represent a surrogate end-point for survival and determining the best way to identify upfront patients who may benefit in this setting [46]. With regard to this, the assessment of candidate biomarkers by mIHC on tumor biopsies prior to initiation of neoadjuvant treatment as well as on post-treatment surgical resection samples may be helpful while preserving tumor architecture to assess complete tumor response. Thus, the mIHC approach could be used to standardize the recently described “Immune-Related Pathologic Response Criteria” in a clinical setting [47]. 

Moreover, another open question that remains to be solved is the use of immunotherapy in special subpopulations, such as elderly patients [48]. Aging is characterized by rebuilding the immune functions, involving both innate and adaptive immunity [49]. By using a brightfield mIHC platform, we recently shown that elderly ≥75 years NSCLC patients have less effective anti-tumor immunoreactivity [33]. While further validation in a larger population is required, our findings suggest that distinct immune pathways may lead to poor outcome in elderly patients with lung adenocarcinoma [33]. Several previous studies demonstrated that the CD4^+^/CD8^+^ ratio may give more prognostic information than either marker alone in solid tumors [50,51,52].

As outlined above, mIHC provides a unique sample-sparing analytical tool to characterize limited clinical tissue samples by multiplexing targets of interest. This method also has the potential to improve clinical diagnostic accuracy and facilitate histopathological interpretation. 

**Figure 3:**
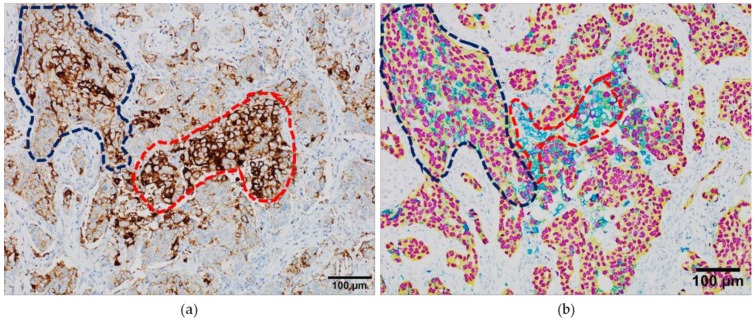
Interpretation of the programmed death-ligand 1 (PD-L1) staining in serial whole-tissue formalin-fixed paraffin embedded samples from a lung adenocarcinoma case. (**a**) PD-L1 expression revealed by conventional immunoperoxidase staining; (**b**) PD-L1 expression revealed by chromogenic multiplexed immunohistochemistry, with the anti-TTF1 antibody colored in purple, anti-AE1/AE3 in yellow and anti-PD-L1 SP263 in teal. Blue dotted line: tumor area; red dotted line, immune cells.

We recently developed in our laboratory (Laboratory of Clinical and Experimental Pathology, Nice, France) two automated brightfield 4-Plex mIHC assays to comprehensively characterize NSCLC major histotypes by multiplexing three conventional IHC markers (e.g., TTF1, p40, AE1/AE3) and three predictive biomarkers (*ALK, ROS1, BRAFV600E*) cleared by the US Food and Drug Administration/European Conformity-*In Vitro* Diagnostic (FDA/CE-IVD) [22]. Some pathology laboratories use chromogenic mIHC on FFPE samples but stain for no more than two markers per tissue slide [45]. The two assays demonstrated no antigenicity loss, steric interference or increased cross-reactivity, providing an analytical tool that can be integrated in a routine clinical workflow [22]. In addition, there are some concerns on the extent to which a multi-color background with color overlap on whole-slide samples could influence the visual interpretation of critical biomarkers. In particular, the PD-L1 expression can be heterogeneous and variably expressed in either tumor or immune cells [53]. By excluding the PD-L1 expressing cells that are unstained with keratin and TTF1 as per tumor-infiltrating immune cells expressing PD-L1, the chromogenic mIHC assay made the visual interpretation straightforward and less ambiguous (Figure 3). 

As the restricted tissue size is a major issue for the management of the vast majority of solid tumors, and individual antibodies rarely demonstrate 100% specificity in the determination of malignancy or cell lineage, a chromogenic mIHC approach with specific multiple protein markers can provide valuable diagnostic information and has the potential to enhance the clinical significance of histological subtyping by delivering substantial prognostic information with therapeutic consequences [54,55]. 

### 3.2. Immunofluorescent Multiplexing

#### 3.2.1. Localization of Immune Cells and Their Relationships with Immunosuppressive Markers in the Tumor Microenvironment

The multiplex immunofluorescence techniques better distinguish the stromal and the tumor compartment and thus have allowed for a more detailed description of the topography of immune cells in cancer. Cruz et al. found that T lymphocytes were predominantly concentered in stromal compartment instead of the epithelial compartment in NSCLC [56]. Based on a quantitative immunofluorescence study, a comparative analysis of the expression of immunosuppressive molecules (e.g., PD-L1, IDO-1, B7H4) with the infiltration of intratumoral cells in lung cancer showed that PD-L1 and IDO-1 were consistently associated with prominent B- and T-cell infiltrates, but B7-H4 was not [57]. This could be explained by the role of IFNγ produced by immune cells in regulating PD-L1 and IDO-1 in the tumor microenvironment [58].

#### 3.2.2. Novel Prognostic Composite Biomarker based on Fluorescence in Situ Multiplexing

One of the first clinical studies based on fluorescent digital pathology was the work of Schalper et al., who reported that the infiltration of intratumoral CD3^+^ and CD8^+^T cells was associated with a better overall survival in lung cancer patients [59]. For the CD8^+^T cell infiltration, this prognostic impact was independent from age, tumor size, histology and stage in multivariate analyses [59]. This technology also allows us to better define the prognostic value of immune cells depending on their localization in the tumor microenvironment. For example, after neoadjuvant chemotherapy, high levels of epithelial but not stromal CD4^+^CD3^+^T lymphocytes correlated with better survival in patients with NSCLC [60].

**Figure 4:**
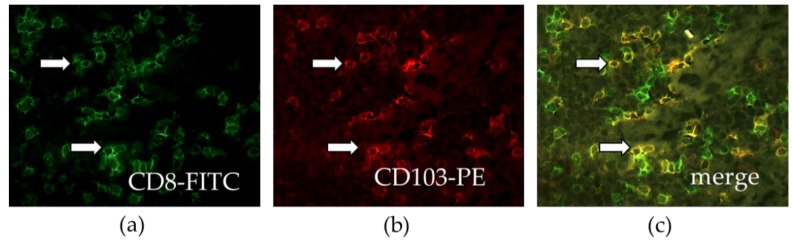
Infiltration of resident memory T cells (CD103^+^CD8^+^T cells) in human lung cancer. Frozen tissue sections derived from lung adenocarcinoma patients were stained by immunofluorescence with antibodies directed against human (**a**) CD8 (green), and (**b**) CD103 (red). (**c**) The co-localization of CD8 and CD103 markers can be detected by merging the mono-stained pictures. The arrows indicate double positive cells. Staining with isotype controls was included for each experiment (20× magnification).

A more complex cell phenotype could also be better characterized with this multiparametric analysis. A novel subpopulation of CD8^+^T cells called resident memory T cells appear to play a major role in immunosurveillance, as they localize in close contact with epithelial tumor cells [61]. They are defined by a composite phenotype including various biomarkers such as CD103, CD49a, CD69 (Figure 4). 

We previously demonstrated that high levels of intratumoral infiltration with a resident memory CD8^+^T cells are associated with a better clinical outcome of NSCLC patients, both in univariate and multivariate analyses [62]. These were a more powerful prognostic marker than the infiltration of total CD8^+^T cells. These data were then confirmed by various clinical studies [63,64].

This technique also allows us to focus beyond just one cell type, and to integrate the relationships that exist between immune cells in the various compartments of tumors and the relative impact of these cellular relationships on the future of patients. For example, a high effector CD8^+^T cell/regulatory T cell ratio in the tumor nest is correlated with a better overall survival than when each cell measured independently [65]. 

#### 3.2.3. Fluorescence Multiplexing Technique to Predict Clinical Response to Immunotherapy

Various parameters such as PD-L1, the expression of PD-1 and the intratumoral infiltration of CD8^+^T cells are considered, especially when combined together, as potential predictive biomarkers of clinical response to immunotherapy [66]. Parra et al., observed higher levels of PD-L1 expression on tumor cells and an increase in the infiltration of T cells and PD-1^+^T cells in the tumor microenvironment of NSCLC after neoadjuvant chemotherapy [60]. These findings confirm studies in other cancers reporting that neoadjuvant chemotherapy, whatever the regimen, makes the tumor microenvironment more permissive to immunotherapy [67,68]. These results suggest that it would be worthwhile to combine chemotherapy and immunotherapy before surgical resection of locally advanced lung cancer.

Using a quantitative multiplex immunofluorescence technique, we reported that *EGFR*-mutated NSCLC weakly expressed PD-L1 and was not infiltrated by CD8^+^T cells suggesting that it would not be prone to respond to immunotherapy [69]. This hypothesis was then clinically confirmed in various clinical trials [70]. Interestingly, we found that a subpopulation of NSCLC displaying chromosomal rearrangement of the *ALK* gene expressed significant levels of PD-L1 on their tumor cells and were infiltrated by PD-1^+^CD8^+^T cells [69]. However, other studies showed that concurrent CD8^+^T cells and high PD-L1 expression on tumor cells tend to be rare in *ALK* positive NSCLCs [71,72]. Clinical trials did not confirm the sensitivity of this cancer subtype to the blockade of PD-1/PD-L1 axis [71]. This may suggest that other resistance mechanisms occur in this population such as the possible co-expression of inhibitory receptors on T cells or the infiltration of immunosuppressive cells [73,74]. Finally, an increase of T cells with a quiescent phenotype defined by a low proliferation and activation status (Ki67 and Granzyme negative) correlated with better overall survival in NSCLC patients treated by anti-PD-1/PD-L1 [75]. Interestingly, in NSCLC patients not treated by immunotherapy, this population of “dormant” T cells did not correlate with a better clinical outcome, supporting the fact that these cells could represent a true predictive biomarker of response to immunotherapy and not a prognostic marker [75]. 

## 4. Image Analysis of Multiplexed Staining

Until recently, pathologic analysis of the IHC signal remained a subjective and time-consuming procedure, wherein the staining intensity, localization and amount had to be manually assessed. Therefore, despite development of practical scoring systems, such as the H-score, the scoring decision is still directly influenced by visual bias [76,77]. Nowadays, with the advent of precision digital immune-oncology, pathologists face a technological transition phase. The convergence of tissue-based mIHC along with automated computer-aided imaging technologies has the potential to make complex information more accessible in routine clinical workflows, improving prognostic and predictive patient stratification [78]. Image analysis and artificial intelligence tools and fields of application in immune-oncology have been outlined in a recent review by Koelzer et al. [78].

The improvement in digital imaging processing systems has opened new doors towards an unbiased, unsupervised, and automatic IHC image analysis by measurement of optical density, which is proportional to the expression extent of specific antigens [77]. Furthermore, application of an automated scoring method for mIHC signals might help pathologists in quantitative comparisons and produce a more accurate characterization of the tumor microenvironment. The mIHC digital image must have the correct stains unmixed into their constituent chromogens for each individual biomarker. Moreover, in order to obtain accurate identification, segmentation and profiling of tumor and immune cells, the mIHC image analysis has to assure the same quantity of chromogen in the color mixture [35]. Several technologies have been developed to decompose each pixel into a collection of constituent signals and the fractions from each of them, in order to convert the whole image into analyte-specific image channels [79]. However, the maximum number of stains that can be unmixed was limited to three, as the linear system had insufficient equations for cases of more than three stains [35]. Alternatively, a novel multi-spectral image deconvolution algorithm has been developed to handle more than three colors and to maintain the biological properties of the protein markers [35]. 

An increasing number of automated digital pathology systems are being used to analyze information from mIHC technology, such as HALO (Indica Labs, London, UK) [80] for up to five colors, Vectra/inForm (PerkinElmer, Waltham, MA, USA) for up to three colors [81], the “Aperio Color Deconvolution Algorithm” or SlidePath (Leica Biosystems, Wetzlar, Germany) for up to three colors [82], BLISS workstation (Bacus Laboratories, Lombard, IL, USA) for up to four colors but restricted to one region-of-interest (ROI), Tissue Studio^®^ 4.0 (Definiens, Munich, Germany) for up to two colors [83], the “Automated Cellular Imaging System” (ACIS III, Dako, Glostrup, Denmark), and Mirax HistoQuant (3DHistech, Budapest, Hungary) [84]. 

**Figure 5:**
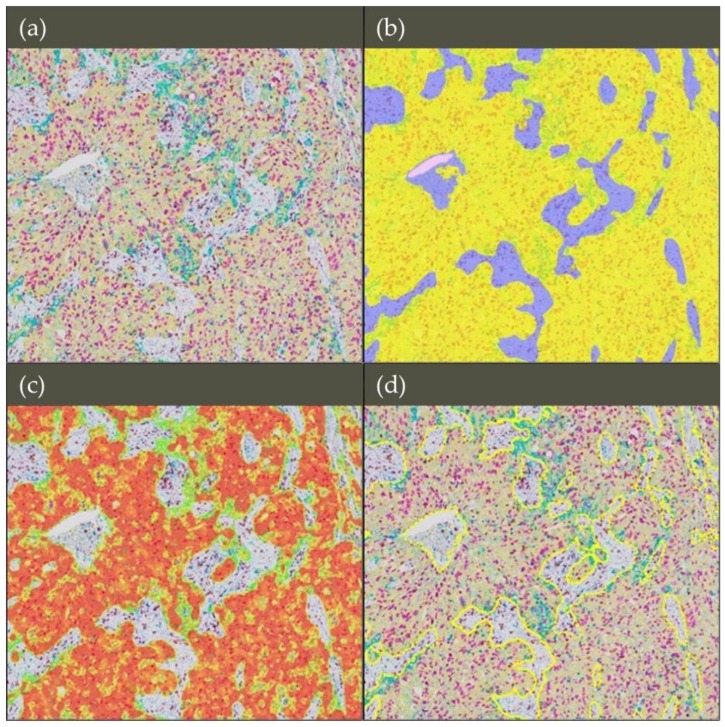
Tissue classification using the random forest classifier in non-small cell lung cancer tissue (20× magnification). (**a**) The multiplexed immunohistochemistry (mIHC) image was scanned with a Nanozoomer 2.0-HT Scanner (Hamamatsu photonics, Hamamatsu, Japan). The stains are as follows: Pan-cytokeratin (yellow), CD8 (brown), Ki67 (purple), PD-L1 (teal) and hematoxylin (dark purple). (**b**) The random forest classifier in HALO was used to separate the image into three classes: tumor, stroma and microscope glass slide. The classifier mask is shown overlaying the mIHC image where classified tumor regions are shown in yellow, stroma regions in purple, and the microscope glass slide in pale pink. (**c**) The probability threshold used by the random forest to detect tumor regions was increased to 70%. A heatmap is displayed where the red regions represent areas most likely to be tumor regions, and the green regions that are less likely. No mask will appear in areas where pixels have below 70% probability of being in the tumor class. (**d**) The classifier to annotations option was used whereby regions can automatically be annotated from the classification mask; only the tumor has been annotated (shown in yellow).

In our own experience we have used HALO, which is an automated quantitative digital pathology platform, compatible with all major microscope/slide scanners and non-proprietary tiff/jpeg formats and allowing for whole-slide and field-of-view analyses. Modules used for mIHC analysis include mIHC (brightfield mIHC), a tissue classifier module for tissue differentiation (e.g., tumor vs. stroma), and a spatial analysis module for interrogating spatial distributions of cell populations within the same, or serial tissue sections. Occasionally, it is critical to separate out the tumor and stroma into two classes, in order to determine the percentage of tumor cells positive for x, versus the percentage of stromal cells positive for x. Manually annotating these regions is extremely laborious and therefore automatic detection of these two regions is required for high-throughput analysis. HALO uses two different machine learning classifiers for automatic tissue detection: the random forest classifier and HALO-AI. The random forest classifier uses the random forest algorithm to assign pixels to a certain class based on color and texture. A random forest classifier is very quick to create and is effective in applications such as differentiating between tumor and stroma as shown in Figure 5. The Serial Section module also allows one to create a classifier on one stain (e.g., an H&E image), and then superimpose the classification onto a registered serial section. Therefore, there is no need to have a tumor marker on each serial section to achieve tumor/stroma separation.

The random forest classifier is quick and easy to set-up but will often suffer when presented with multiple variable tissue staining; such is often true for large clinical cohorts. In such situations HALO-AI, a deep learning classifier can be used. HALO-AI is a convolutional neural network for pattern recognition within a tissue section. Whilst a pathologist’s input is increased relative to random forest, the training results in a highly robust classifier that can be used across large cohorts. HALO-AI can even be trained to recognize different tissue classes across different stains. The probability map and conversion to annotation features can also be used in HALO-AI. 

**Figure 6:**
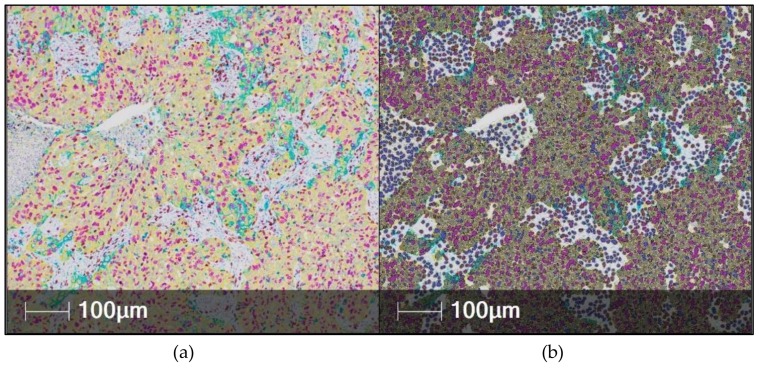
Automated digital analysis of multiplexed immunohistochemistry (mIHC) using the HALO software in non-small cell lung cancer tissue. (**a**) The mIHC image was scanned with a Nanozoomer 2.0-HT Scanner (Hamamatsu photonics, Hamamatsu, Japan) using 20x magnification. The stains are as follows: Pan-cytokeratin (yellow), CD8 (brown), Ki67 (purple), PD-L1 (teal) and hematoxylin (dark purple). (**b**) The HALO mark-up image shows colors similar to the original stain color and in the same cell compartment (nucleus/cytoplasm/membrane as the stain is found. The user can select different colors to be used in the mark-up image if they wish.

Once the selected classifier has been created and saved, it can then be used in the mIHC analysis in HALO. In brightfield, the mIHC module allows the pathologist to detect up to 5 stains, including an exclusion stain, in any cell compartment (nucleus, cytoplasm, membrane). The exclusion stain option can be used to exclude tar within lung tissue. An example of a mIHC analysis in HALO is shown in Figure 6. 

Prior to running the mIHC analysis, pathologists can define specific phenotypes such as active T cells (e.g., dual-positive cells for brown and purple stains will be identified as dual positive for CD8 and Ki67; Figure 6). 

**Figure 7:**
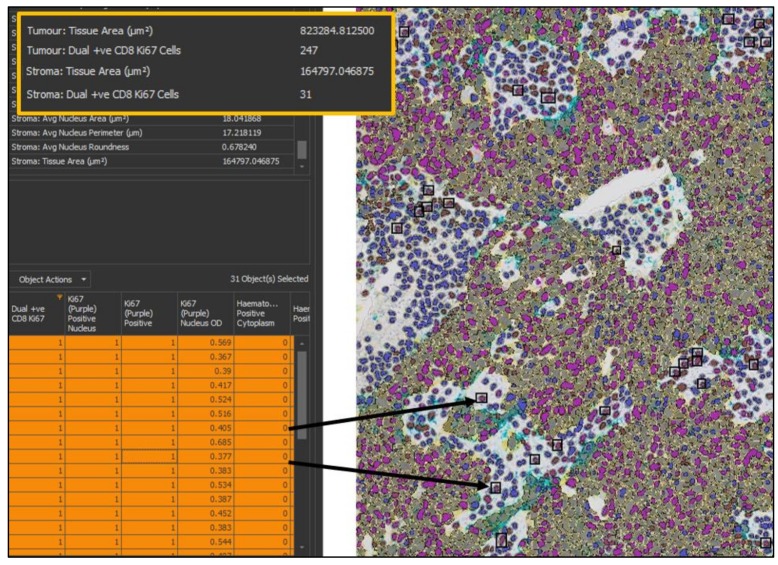
Results of a multiplexed immunohistochemistry (mIHC) analysis in HALO. The top left table provides the summary results from the analysis; important outputs in this analysis are the density of cells co-expressing both CD8 and Ki67 in the tumor and stroma, and so the data relating to this has been highlighted. The bottom left table is HALO’s interactive cell-by-cell data table, which can be mined to find specific cell types. Here, only cells that are positive for CD8 and Ki67 and are in the stroma have been selected. HALO will find the cells selected in the image viewer (right, 20× magnification) by putting a black box around each cell.

When this is run in conjunction with the pre-made classifier, information about the number of cells with a specific phenotype in both the tumor and the stroma can be obtained. Additionally, HALO’s interactive cell-by-cell data table allows easy localization of the phenotyped cells on the image. In the example analysis in Figure 7, outputs will include those for the entire image, those specific to the tumor and those specific to the stroma. Other outputs include the number of cells positive for each stain in each compartment, number of cells with different stain co-localizations, the average optical density values for each stain in each compartment, cell/nucleus/cytoplasm/membrane area, and tissue areas in square microns.

After running a mIHC analysis in HALO, the pathologist then has the option to generate spatial information using the spatial analysis module. As outlined above, spatial information is becoming increasingly important in cancer research, prominently in the immune-oncology field [36,85]. Three different types of spatial analysis can be performed in HALO: nearest neighbor, proximity analysis and invasive margin analysis. Nearest neighbor outputs will calculate the average distance of two cell populations based on their nearest neighbors. Proximity analysis allows you to calculate the number of cells of one phenotype (e.g., CD8+ cytotoxic T lymphocytes) within a defined distance of another cell type. Lastly, the invasive margin analysis allows you to count the number of cells within a user defined distance of the invasive margin. 

Similarly, the HALO image analysis software was recently used to demonstrate the divergent state of exhaustion of the PD-1 receptor in T cells with impaired effector cytokine production, while producing CXCL13, which mediates immune cell recruitment to tertiary lymphoid structures [80]. Importantly, the presence of PD-1^high^ cells was strongly predictive for both response and survival in a cohort of NSCLC patients treated with a PD-1 blocking agent [80].

In the immunofluorescence multiplexing field, the use of scanners (fluorescent or spectral) represents a major technological advance by enabling the utilization of multiple and sometimes unstable fluorochromes (e.g., phycoerythrins) and thus more than 7 different antibodies on the same slide. For example, Vectra^®^ systems or Polaris® (PerkinElmer) allow the capture of information by spectral resolution in the visible and in the near infrared band (bandwidth between 420 and 900 nm). Vectra^®^ or Polaris^®^ allows extremely precise quantitative (cell-by-cell) management of the markings of different tissue samples, in brightfield or fluorescence detection. Detection and phenotypic characterization of cells in tissues, combined with bioinformatic image analysis is possible thanks to the InForm^®^ software (PerkinElmer). This software allows automatic analysis of parameters that cannot be accurately discerned by the human eye (cell forms, multiple molecule networks, vascular network). 

**Figure 8:**
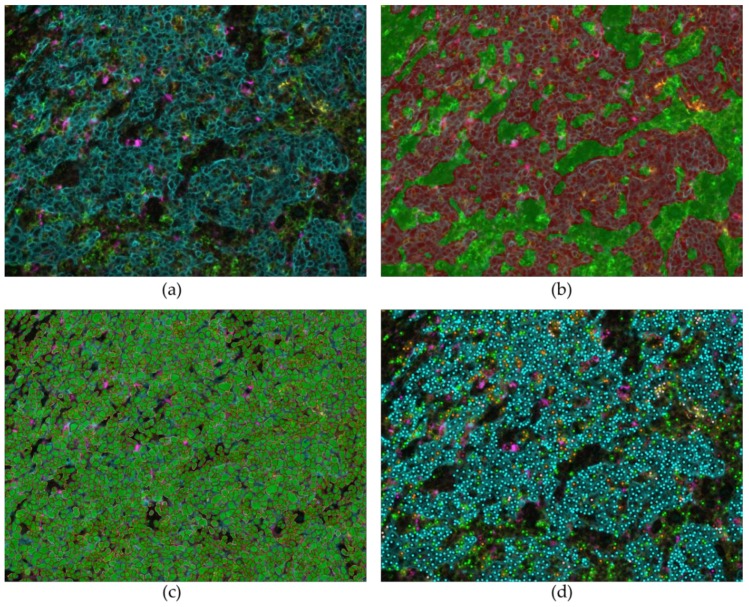
Automated digital analysis of fluorescent multiplexing using Inform software in tonsil tissue (20× magnification). (**a**) Multiparametric fluorescent staining Pan-Cytokeratin (turquoise), CD4 (green), CD68 (purple), PD-1 (red), PD-L1 (yellow) and dapi (blue). (**b**) Tissue segmentation: identification and recognition of tumor areas (red) or stroma (green). (**c**) Individual cells identification and segmentation, with nuclear, membranous and cytoplasmic segmentation. (**d**) Phenotyping: identification of the cells on the slide, with their phenotypes, among all the cells present in the image, or among the cells stained.

Franchising of autofluorescence by the "Autofluorescence Reduction Technology” (ART™, PerkinElmer) technique is possible with the Inform^®^ software (PerkinElmer). Of course, the technologies developed for a specific type of cancer are subsequently transposable to the majority of other tumor proliferations or inflammatory diseases. Finally, virtual slides can be analyzed automatically (cell counting, surface measurements, etc.) using dedicated image analysis software (Figure 8). In particular, some software enables the quantification of weakly expressing and overlapping biomarkers within cells and cellular compartments.

These new approaches allow us to explore cellular interactions to find biomarkers in a non-supervised manner. The education of the software remains long and tedious, with a phase of learning or "teaching". However, an approach without *a priori* knowledge can also be developed in parallel. Several companies developed such software (e.g., Definiens AG, Munich, Germany; TRIBVN Healthcare, Châtillon, France; Owkin, Paris, France; Imstar, Paris, France; Indica Labs; PerkinElmer). These software systems are becoming more and more efficient, and they can differentiate anatomical structures, such as glands [86], but the recognition of cell units is more delicate. The results obtained in the context of cross-sectional research studies are, however, very impressive and we must expect a change in diagnostic habits with the implementation of deep learning [87].

Finally, an important issue for mIHC digital analysis and relevant data extraction is the calibration of the signal acquisition technology and the control of variations caused by the different staining techniques when several batches are required to analyze large clinical series (e.g., for biomarker validation). These controls are also necessary for the valid comparison of different series or studies and ultimately for clinical application [88].

## 5. Advantages and Current Limitations of Multiplexed Immunohistochemistry

Recently developed multiplexing platforms exhibit compelling advantages. The major advantage of mIHC, which may also warrant its implementation in the routine clinical workflow, is related to maximal data harvesting per tissue section, improvement in the quality and detail of pathology analysis and efficient tissue utilization, which is crucial when the availability of sample is limited [89]. Approaches like mIHC enable pathologists to gather a wealth of data from a limited amount of tissue. This is especially promising for NSCLC patients whose tumors are in a difficult-to-access location, where only a small needle or cytology sample can be obtained. It also enables more research to be conducted with less material than is often required [89]. Unlike other multiplex approaches, such as next generation sequencing or mass spectrometry, mIHC gives an edge to analyze co-expression and to quantify single-cell expression with the spatial relationships of many targets while preserving tissue integrity. Several studies have shown that the proximity of certain immune cells within a tumor microenvironment correlates with patient outcome [41,85,90].

Recently developed strategies in the field of brightfield chromogenic mIHC have enabled automation of mIHC assays through the use of commercially available primary antibodies with their respective anti-species secondary antibody to ensure staining reliability and reproducibility, toward the clinical application [22]. Moreover, conventional brightfield microscopes and scanners can accommodate image acquisition of the stained slides [78]. 

However, multiple pre-analytical and analytical challenges arise when using chromogens for high-level mIHC analysis. The limited number of available chromogens, compared to highly multiplexed fluorescent assays, limits the degree of flexibility for biomarker research. As chromogenic mIHC is technically similar, in some ways, to conventional IHC it is subjected to the same critical hurdles [91]. The lack of standardization due to pre-analytic variables, including fixation time, type of fixative, dehydration, clearing, paraffin impregnation, and drying and storage of the slides, still represents a major potential challenge [92]. Similarly, poorly characterized or cross-reactive antibodies will give non-reproducible results [93]. For instance, despite numerous efforts to standardize the IHC markers used in breast cancer (ER/PR/HER2), they still demonstrate significant inter-laboratory and intra-laboratory variability [94]. If such issues cannot be overreached for these “conventional” IHC biomarkers, the multiplexing of several markers will need sufficient robustness prior to a clinical use. As for the clinical single IHC assays, a positive tissue control previously validated and characterized should be run on each same slide tested with mIHC. This would allow “real-time” validation of the multiplexed staining along with the quality control of data generated by the mIHC assay. 

As tumors frequently harbor significant cellular and spatial heterogeneity (e.g., stroma, tumor-stroma interface, intratumoral), in particular for immune markers such as PD-L1 or CD8 infiltrates [95], it is essential to perform high-resolution multiplexed analysis across whole tumor sections. It has been demonstrate that the analysis of small ROIs generates significant variation and errors in the assessment of tumor and immune markers in cancer [96,97]. Hence, there is a need for integrated mIHC systems enabling high-degree of multiplexing coupled with digital analysis for high-resolution analyses on whole tumor slides [98].

Moreover, mIHC is the only technology enabling quantitative information on multiple distinct subtypes of tumor-infiltrating immune cells within a preserved tissue architecture, hence allowing the analysis of the topology and proximity between specific cell populations [99]. Ultimately, the quantitative spatial profiling of key tumor-immune pathways could improve the stratification of cancer patients for immunotherapy [100]. In addition, the explosion of potentially important or actionable biomarkers poses both cost and selection challenges. The increase in the number of developed chromogens could make this challenge somewhat easier to handle [16]. However, the current cost of the primary antibodies or different chromogens and the instrumentation requirements are still high. More than four antibodies can be sequentially incubated on autostainers, reducing the difficulty, delay and therefore cost to perform the mIHC analysis in a clinical setting, although, as noted above, pre-analytical variability and antigen retrieval methods will first need to be critically evaluated. Moreover, evaluation of multiple targets per tissue slide will require digital image viewing with analysis tools for computer-assisted interpretation that are yet to be readily integrated in the clinical workflow [78]. For a wide clinical implementation and pathologists’ acceptance, regulatory and reimbursement rules should be planned in the near future. Nevertheless, the extraordinary value of such a technological approach to improve pathology interpretation and to yield new insights into understanding cancer phenotypes with direct clinical impacts warrants further effort. 

The different considerations presented above could be declined for the fluorescent mIHC. The specificity of the staining has been improved with the use of tyramide techniques allowing simultaneous staining with 7 to 9 colors in a same slide. The different technical implementations described in this article have to reinforce the efforts made to increase the knowledge about microenvironment. Fluorescent staining keeps an advantage in research for the observation of very rare events, rare cells, co-localization and still allows a better study of the different cell compartments. Nevertheless, this technique is still difficult to be used in routine; the signal reproducibility is difficult to be obtained, even with an automation of the staining.

Several alternative multiplexed technologies for a use on FFPE samples have recently been developed (e.g. multiplexed ion beam imaging-MIBI, IONpath, Inc., Menlo Park, CA, USA; imaging mass cytometry, Fluidigm, South San Francisco, CA, USA; digital spatial profiling technology, NanoString Technologies, Inc., Seattle, WA, USA; InSituPlex, Ultivue, Cambridge MA, USA) demonstrating a high degree of multiplexing, and could be complementary to mIHC approaches described herein [89,101,102,103]. 

## 6. Conclusions

Technological advances in mIHC and the introduction of automated slide scanners has allowed for huge amounts of data to be generated in a single experiment. Combining this with automated digital analysis means the data can be analyzed in a quantitative and efficient manner, producing a high-throughput workflow for molecular and immune profiling with the promise of discovering novel biomarkers and improving clinical management of patients with NSCLC.
